# Molecular evolution of anthocyanin pigmentation genes following losses of flower color

**DOI:** 10.1186/s12862-016-0675-3

**Published:** 2016-05-10

**Authors:** Winnie W. Ho, Stacey D. Smith

**Affiliations:** Department of Ecology and Evolutionary Biology, University of Colorado, Boulder, USA

**Keywords:** Molecular decay, Reversibility, Trait evolution, Predictability, Gene fate, Evolutionary trajectory

## Abstract

**Background:**

Phenotypic transitions, such as trait gain or loss, are predicted to carry evolutionary consequences for the genes that control their development. For example, trait losses can result in molecular decay of the pathways underlying the trait. Focusing on the Iochrominae clade (Solanaceae), we examine how repeated losses of floral anthocyanin pigmentation associated with flower color transitions have affected the molecular evolution of three anthocyanin pathway genes (*Chi*, *F3h*, and *Dfr*).

**Results:**

We recovered intact coding regions for the three genes in all of the lineages that have lost floral pigmentation, suggesting that molecular decay is not associated with these flower color transitions. However, two of the three genes (*Chi*, *F3h*) show significantly elevated dN/dS ratios in lineages without floral pigmentation. Maximum likelihood analyses suggest that this increase is due to relaxed constraint on anthocyanin genes in the unpigmented lineages as opposed to positive selection. Despite the increase, the values for dN/dS in both pigmented and unpigmented lineages were consistent overall with purifying selection acting on these loci.

**Conclusions:**

The broad conservation of anthocyanin pathway genes across lineages with and without floral anthocyanins is consistent with the growing consensus that losses of pigmentation are largely achieved by changes in gene expression as opposed to structural mutations. Moreover, this conservation maintains the potential for regain of flower color, and indicates that evolutionary losses of floral pigmentation may be readily reversible.

**Electronic supplementary material:**

The online version of this article (doi:10.1186/s12862-016-0675-3) contains supplementary material, which is available to authorized users.

## Background

Understanding the predictability of the molecular changes associated with phenotypic transitions is a major goal in evolutionary biology. Evolutionary genetic studies across a wide range of organisms and traits have revealed that repeated phenotypic transitions are often caused by similar changes at the genetic level [1, 2]. In some cases, this molecular convergence extends not only to the locus but also to the type of mutation and even its nucleotide position [3–5]. These patterns suggest that the causes of phenotypic evolution may often be predictable at the molecular level, given knowledge of the genetic basis for the trait [6, 7]. Indeed, for many traits, the mutations responsible for convergent phenotypes are concentrated in a small set of “hotspot” loci [8] and are restricted to particular classes of mutations (e.g. [9, 10]).

In addition to predictability in the mutations that cause phenotypic transitions, the genetic changes that follow phenotypic transitions can also be predictable. For example, transitions to a parasitic or symbiotic habit are often followed by pseudogenization and molecular decay in particular functional gene categories [11, 12]. Even genes which are conserved for other functions may experience an increase in evolutionary rate if the trait transition is associated with reduced breath or level of expression [13, 14]. Predictable molecular patterns are also seen at the protein level, where substitutions that cause changes in function may be followed by compensatory mutations to offset fitness declines [15, 16], or by mutations to optimize the derived activity and/or increase stability of the new conformation [17–20]. Despite having strong expectations about the genetic changes that are likely to follow many types of phenotypic transitions [21, 22], relatively few studies have tested these predictions at macroevolutionary scales [23, 24].

Because trait losses occur relatively frequently across the tree of life (e.g. [25, 26]), this type of transition provides a powerful tool for understanding the predictability of genetic changes following phenotypic evolution. Trait losses may lead to a range of outcomes for genes in pathways underlying those traits. One possible outcome is the gradual erosion of pathway genes through the accumulation of inactivating mutations, leading to pseudogenization or even gene loss [27, 28]. This process is expected to occur over the span of 0.5 to 6 million years [29] and has been documented in association with many instances of trait loss, such as the loss of eyes in cavefish [30] and teeth in edentulous mammals [31]. However, molecular decay may be prevented if the underlying genes are involved in the development of multiple traits beyond the trait that was lost. In this case, the genes may experience stabilizing selection to maintain their function or positive selection to improve function in those other contexts [29, 32]. Another possible outcome for pathway genes following trait loss is recruitment for an entirely new function, although this pattern has never been documented. To some extent, these possible evolutionary trajectories for genes after trait loss parallel those following gene duplication, in which new gene copies may undergo pseudogenization, subfunctionalization, neofunctionalization, or escape from adaptive conflict [32–34]. Both evolutionary scenarios (trait losses and gene duplications) share a reduction in constraint that alters the selective forces experienced by genes, and consequently, their evolutionary fates. However, compared with the literature on gene duplication (reviewed in [35]), much less attention has focused on the molecular fates of single-copy genes associated with cases of trait loss.

**Fig. 1:**
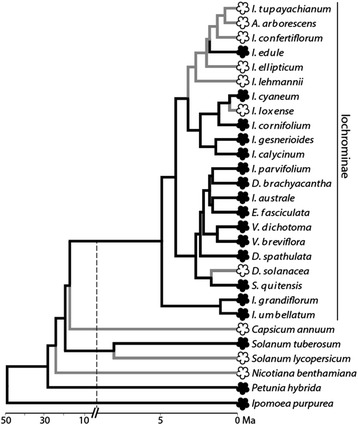
Distribution of floral anthocyanin pigmentation across Iochrominae. Lineages with pigmentation are represented with black flowers and black lines; those without are represented with white flowers and gray lines. Generic names within Iochrominae are abbreviated as follows: *A.* = *Acnistus*, *D.* = *Dunalia*, *E.* = *Eriolarynx*, *I.* = *Iochroma*, *S.* = *Saracha*, and *V.* =*Vassobia*. Phylogeny and estimated divergence times from Sarkinen et al. [39] and Muchhala et al. [86]. Note that the timescale is split to accommodate the shallow divergences within Iochrominae as well as the deeper splits among the outgroups

Here we examine the molecular evolution of pigmentation pathway genes following repeated losses of floral pigmentation, focusing on the Andean clade Iochrominae in the tomato family, Solanaceae. This clade of roughly 35 species is well known for its diversity of floral colors [36], and like many other angiosperms, these colors largely derive from red, purple, and blue anthocyanin pigments [37]. These pigments are primarily produced in flowers, with little to no detectable presence in vegetative tissue in most taxa [38]. Over the estimated 5 million year history of Iochrominae [39], at least three lineages have lost floral anthocyanin pigmentation in association with transitions from the ancestral state of purple flowers to white or yellow flowers (Fig. 1, [40, 41]). The oldest loss (ca. 3 mya) is associated with a clade that has subsequently diversified to give rise to seven taxa (five shown in Fig. 1), one of which has regained pigmentation. The other two losses represent more recent events that occurred in single species (*I. loxense* and *D. solanacea*). The time span of these transitions (ca. 0.5 to 3 mya) encapsulates the range over which we would expect to see molecular consequences of changes in selective constraint on pathway genes following trait loss [29, 30]. If selection maintained these genes primarily for their role in floral pigmentation, we expect losses of pigmentation to be followed by relaxed selective constraint and possibly pseudogenization. Alternatively, if the decay of these genes carries negative pleiotropic consequences, either for anthocyanins in other tissues or for the production of related compounds, the genes might remain under purifying selection, even in white-flowered lineages. Finally, if releasing genes from their role in floral pigmentation allows them to adopt new functions, then we might expect evidence of positive selection.

**Fig. 2:**
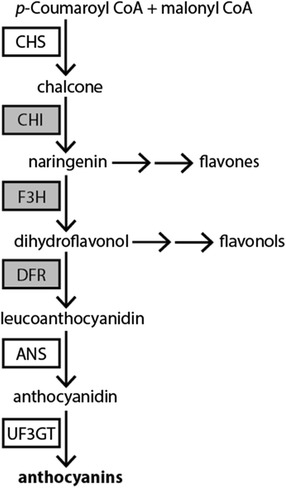
Core anthocyanin biosynthetic pathway adapted from Rausher [56]. Enzymes include: CHS, chalcone synthase; CHI, chalcone isomerase; F3H, flavanone-3-hydroxylase; DFR, dihydroflavonol reductase; ANS, anthocyanidin synthase; and UF3GT, UDP-glucose flavonoid 3-O-glucosyl transferase. Several of the steps required for anthocyanin pigment production are shared with other uncolored flavonoid compounds (e.g., flavones and flavonols). The enzymes examined in this study are shaded in gray

In order to test these possible effects of trait loss on molecular evolution, we focus on a set of three anthocyanin pathway genes, *Chi* (chalcone isomerase)*, F3h* (flavanone-3-hydroxylase)*,* and *Dfr* (dihydroflavonol-4-reductase)*.* The *Dfr* gene represents the most downstream step among these three, and it appears to be exclusively involved in anthocyanin biosynthesis in Solanaceae ([42], Fig. 2). Thus, it might be predicted to experience the strongest effects of relaxed constraint following loss of floral anthocyanins. By contrast, the other two upstream enzymes are required for the production of multiple flavonoids, including anthocyanins, flavones, and flavonols (Fig. 2). The uncolored flavones and flavonols are primarily involved in responses to UV stress [43, 44], but also play a role in male fertility and signaling in some species [45, 46]. Thus, *Chi* and *F3h* might remain under purifying selection despite the loss of floral pigmentation because of their pathway position and functional significance. The dynamics of *Chi* evolution may also be influenced by the fact that, unlike the single copy *Dfr* and *F3h*, this enzyme is encoded by two loci (*Chi-A*, the principally active copy, and *Chi-B*, only expressed in young anthers) [47, 48]. In this analysis, we focus on the *Chi-A* copy (hereafter ‘*Chi*’) that is required for floral pigmentation in Solanaceae [48, 49]. The extent to which these genes experience relaxed selective pressures following the loss of floral pigmentation has important consequences for the evolutionary trajectory of these plant lineages, as the molecular decay of any of these loci would significantly reduce the potential for future regain of this trait.

## Results

### Sequence variation in *Chi*, *F3h*, and *Dfr* across Iochrominae

We recovered complete coding regions for the three anthocyanin pathway genes *Chi* (717 bp), *F3h* (1113 bp), and *Dfr* (1179 bp) from all of the sampled pigmented and unpigmented Iochrominae (Fig. 1). Within Iochrominae, we observed no length variation (insertion-deletion events). Across all loci, pairwise divergence was low, with less than 3 % difference at the nucleotide level and less than 1.5 % at the amino acid level across Iochrominae. Divergence was higher at the family level, ranging from 8 % at the amino acid level in *Dfr* across Solanaceae to 20 % in *Chi*, the most rapidly evolving of the three genes (Additional file 1: Figure S1). However, we did not observe any clear inactivating mutations, such as premature stop codons, or frameshifts, in any of the sequences (Additional file 2: Table S1).

### Variation in selective constraint across genes and across lineages

**Fig. 3:**
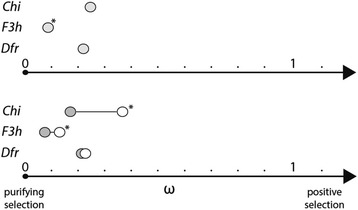
Patterns of variation in the dN/dS ratio (ω) across genes and between pigmented and unpigmented lineages. The upper panel shows ω values for the single ratio model for each of the three loci (see also Additional file 4: Table S3). * represents a locus with a significantly lower ω value than the other two loci. The lower panel shows ω values for pigmented (dark gray dot) and unpigmented (white dot) lineages under the two-ratio model. * represents cases in which the unpigmented lineages have significantly higher ω values

We applied codon-based maximum likelihood methods to characterize patterns of molecular evolution across these loci and test for the effect of pigment loss on selective constraint. Models with a single ratio of nonsynonymous to synonymous substitution rates (dN/dS or ω) for each locus resulted in values between 0.09 and 0.24, suggesting that these loci have predominantly experienced purifying selection (Additional file 3: Table S2a, Fig. 3). Among the three loci, *Chi* has the highest dN/dS ratio, followed by *Dfr* and *F3h*. The values for *Chi* and *Dfr* are statistically indistinguishable, while that for *F3h* is significantly lower (Additional file 4: Table S3, Fig. 3). The range of dN/dS ratios for these genes in our study is similar to that found for anthocyanin transcription factors and other core anthocyanin pathway genes in previous studies [50, 51].

We next estimated branch models with separate dN/dS ratios for pigmented and unpigmented lineages in order to test the hypothesis that these flower color transitions have altered the selective pressures acting on pigmentation genes. The two-ratio models revealed higher dN/dS ratios in unpigmented lineages for all of the loci (Fig. 3), consistent with relaxed selective constraint. The ratio for unpigmented lineages was nearly twice as high as that for pigmented lineages in both *Chi* (0.377 vs. 0.185) and *F3h* (0.128 vs. 0.0792), and the two ratio models for these genes resulted in significantly higher likelihoods than the single ratio models (Additional file 3: Table S2a). Although dN/dS is slightly elevated in unpigmented lineages for *Dfr*, a single ratio model could not be rejected (Additional file 3: Table S2a).

Considering that the timing of losses of floral pigmentation varies across the sampled taxa (Fig. 1), we examined two additional models allowing for different ratios among the unpigmented lineages. In the first of these, we divided the unpigmented lineages between those in the outgroups (three taxa) versus those in the ingroup (seven taxa). We found the same pattern as in the two ratio models; the unpigmented lineages in both the ingroup and outgroup have elevated dN/dS ratios relative to the background ratio in the pigmented lineages for both *Chi* and *F3h*. However, this division did not represent a significant improvement over the two ratio models (Additional file 3: Table S2a). We also tested models separating the clade which has diversified since a recent loss (comprising *I. tupayachianum*, *A. arborescens*, and *I. confertiflorum)* from the remaining unpigmented lineages and recovered the same result (elevated dN/dS ratios in unpigmented lineages but no improvement in likelihood over the two ratio models). These analyses suggest that the pattern of elevated dN/dS ratios following losses of floral anthocyanins is not taxon-specific (e.g., only in Iochrominae) but rather shared across all of the sampled lineages without floral anthocyanins.

### Testing for positive selection in unpigmented lineages

The finding of elevated dN/dS ratios following losses of floral pigmentation could be attributed either to reduced selective constraint or positive selection acting on multiple sites across the genes. We implemented an additional series of maximum likelihood models to distinguish among these explanations. First, we fit alternative branch-site models to test the hypothesis that a proportion of sites within the unpigmented lineages has experienced positive selection (dN/dS greater than 1). In these comparisons, we found no evidence of positively selected sites in the unpigmented lineages (Additional file 3: Table S2b). The majority of the sites (66 to 88 % depending on the locus) are inferred to have experienced purifying selection in both pigmented and unpigmented lineages while the remainder are evolving neutrally in one or both (Additional file 3: Table S2b). As additional confirmation of this pattern, we fit simpler sites models (in which the categories are not partitioned across pigmented and unpigmented branches), and these analyses were also consistent with a lack of positive selection. Models including a proportion of sites under positive selection did not result in a significantly higher likelihood for *Chi* or *F3h* (Additional file 3: Table S2c). Although the addition of a proportion of positively selected sites did significantly improve the likelihood for *Dfr*, no positively selected sites were identified in the using the Bayes Empirical Bayes analysis (*p* > 0.95) (Additional file 3: Table S2c). Given previous work on *Dfr*, we suspect the better fit of models with a category of positively selected sites reflects selection associated with shifts among different pigment types (e.g., blue and red: [19]) as opposed to gains and losses of pigments.

Because the dN/dS based tests may have low power when selection has not acted repeatedly on the same sites [52], we employed alternative methods that focus on physiochemical properties [53]. Unlike dN/dS based tests, which treat all non-synonymous substitutions equally, these methods consider the physiochemical effects of amino acid changes and the magnitude of their predicted impacts on protein function. Positively selected sites are identified by comparison with a expected distribution of effects based on codon frequencies [54]. Focusing on the unpigmented lineages, we found zero substitutions with significant physiochemical effects in *F3h* (Additional file 5: Table S4). Only one such substitution was identified in *Dfr*, and this change occurred in one of the two unpigmented outgroup taxa (Additional file 5: Table S4). We found six sites with non-conservative substitutions in unpigmented lineages in *Chi* (Additional file 5: Table S4) although a far greater number (22 total) were found among the pigmented lineages (Additional file 5: Table S4). Thus, the proportion of radical changes on unpigmented lineages (6 of 28, or 0.21) is less than the proportion of unpigmented branches in the phylogeny (15 of 54, or 0.28). These patterns suggest that positive selection is not elevated in the unpigmented lineages compared to pigmented ones, but that overall, *Chi* has experienced a more dynamic evolutionary history than the other two loci, as evidenced by its longer branch lengths (Additional file 1: Figure S1).

## Discussion

Following evolutionary losses of floral anthocyanin production, we predicted a range of possible outcomes for genes in the anthocyanin pathway. On one extreme, if the role of these genes was limited to flower color, we would predict that losses of floral pigmentation would lead to relaxed constraint and possibly pseudogenization. However, to the extent that these genes are important for traits beyond flower color, we might expect them to experience purifying selection, even in lineages without floral anthocyanins. In addition, the genes could undergo positive selection to optimize their non-floral functions or to acquire new functions in lineages which no longer produce floral anthocyanins. Our results indicated that all three of the pathway genes remain under purifying selection and have not experienced positive selection following multiple losses of pigmentation across the phylogeny. While the ratio of non-synonymous to synonymous substitutions was elevated in lineages without floral anthocyanins, we did not find evidence for positive selection, as would have been anticipated if the genes had shifted to new functions. These results suggest that while losses of flower color relax the level of purifying selection acting on pigment genes, they do not lead to irreversible molecular decay.

### Conservation of anthocyanin pathway genes

Our study supports previous findings that the core genes of the anthocyanin pathway are highly conserved, likely due to their wide range of functions in plant physiology. Anthocyanins and related flavonoids are found across all land plants and thus, the structural elements of the pathway trace back to their common ancestor over 400 million years ago [55, 56]. Depending on the taxon, these compounds may play a role in pigmentation, UV stress response, signaling, and defense [37, 43, 46]. Accordingly, a loss of function of any of the core genes of the pathway (Fig. 2) would likely affect many aspects of plant fitness. Consistent with this hypothesis, we found that *Chi*, *F3h*, and *Dfr* have remained conserved through multiple rounds of flower color loss at varying timescales (Fig. 1). All of the white and yellow flowered lineages presented complete coding regions, without premature stop codons or frameshift mutations, suggesting that these copies have retained their function. Although additional experiments would be required to confirm their function (e.g. [21, 57]), the presence of uncolored flavonoids (e.g., flavonols and flavones, Fig. 2) in the flowers of several of the unpigmented taxa [38, 58] supports the conclusion that *Chi* and *F3h* have been conserved. The factors responsible for the conservation of *Dfr* are less clear as it is only known to function in anthocyanin biosynthesis in Solanaceae [42] and anthocyanins have thus far not been detected in non-floral tissues in the white and yellow-flowered lineages of Iochrominae [38]. One possibility is that *Dfr* contributes to pigment production in these lineages but only under particular conditions (e.g., drought or heat stress; [43]). Alternately, this enzyme may participate in additional biochemical reactions beyond those presently identified in the literature (Fig. 2).

Although this represents the one of the few systematic studies of anthocyanin genes across a clade (see also [50]), the strong conservation of *Chi*, *F3h*, and *Dfr* is consistent with findings from population level studies. Decades of research on flower color variation in natural populations have failed to uncover any segregating loss-of-function mutations at *Chi* or *F3h* and only two for *Dfr* [59, 60]. Moreover, the individuals carrying the known *Dfr* mutations in both cases are exceptionally rare relative to their pigmented counterparts. Given that such loss-of-function mutations are known to be quite common among horticultural varieties, these patterns at micro and macroevolutionary scales suggest that selection strongly disfavors mutations which disrupt the function of any of the core genes, preventing their fixation [51].

The conservation of these core genes stands in contrast to other elements of the larger flavonoid pathway, which have the potential to rapidly pseudogenize. In particular, the *F3’5’h* gene, which hydroxylates dihydroflavonols to produce the precursors of blue anthocyanins, has been found to repeatedly experience pseudogenization or deletion following transitions to red flower colors [61, 62]. In both well-studied cases, these color transitions have been associated with floral radiations occurring on the order of 1 to 2 million years ago [36, 39, 63]. Compared to the core genes examined in this study, which are required for production of anthocyanins and many other flavonoids, the role of *F3’5’h* appears to be limited to the blue anthocyanins, lessening the potential pleiotropic effect of its loss [21]. This contrast between the evolutionary history of *F3’5’h* and the core genes examined here suggests that the timespan of the Iochrominae radiation (ca. 5 million years) would be sufficient to observe pseudogenization of *Chi*, *F3h*, or *Dfr* if these genes were not maintained by selection.

### Relaxed constraint following losses of floral pigmentation

Although the core genes have been conserved in lineages without floral anthocyanins, our analyses indicate that *Chi* and *F3h* have experienced altered selection pressures. Models allowing different dN/dS ratios for pigmented and unpigmented branches of the phylogeny resulted in a significantly better fit to the data (Additional file 3: Table S2a), and in both cases, the ratio for the unpigmented branches was roughly twice the ratio for the pigmented branches (Fig. 3). Even with this increase, all of the dN/dS values fall into the range expected for genes that are under purifying selection (i.e., less than 1). For *Dfr*, we also found an increase in dN/dS in unpigmented lineages, but the addition of this extra parameter did not improve the likelihood (Additional file 3: Table S2a).

While such an elevated dN/dS could be due to the action of positive selection, our subsequent analyses suggest that instead the pattern is due to relaxed selective constraint. Branch-site models that allow a proportion of sites to experience positive selection (dN/dS > 1) did not provide a better fit to the data and identified no positively selected sites in unpigmented lineages (PP > 0.95). Given that these analyses do not account for the potential functional consequences of mutations, we compared these results to an alternative physiochemical approach to identifying positive selection [53]. These analyses found little evidence of radical amino acid substitutions in *Dfr* and *F3h*, and none within the white or yellow flowered Iochrominae. While *Chi* experienced a larger number of radical changes, most of these occurred in pigmented lineages, and in particular, in the long branches of the outgroups (Additional file 5: Table S4). Thus, although some functional evolution has likely occurred across the phylogeny, these analyses do not implicate losses of flower pigmentation in driving these changes.

As with our study, many instances of trait loss have been followed by relaxed selection on the underlying genes (e.g. [30, 64]), while positive selection on these genes has never been reported. Theoretically, once a gene is no longer required for a particular task, it has the potential to be co-opted for a new function, in a similar fashion to what has been observed for duplicate copies of genes (e.g., [32, 65, 66]). One explanation for the lack of empirical examples may simply be the relative paucity of traits for which the genetic basis is sufficiently well known to test the effects of trait loss on molecular evolution of the underlying pathway. We suspect that cases may be uncovered as our knowledge of the genotype-phenotype map expands to include a wider array of traits and taxa.

### Pathway position and rate variation

Studies of biochemical pathways have shown that properties of the interacting genes often predict patterns of molecular evolution. For example, more highly connected enzymes (e.g., those share more substrates or products with other enzymes) tend to evolve more slowly than those which are less connected ([67], but see [68]). In the case of the anthocyanin pathway, the upstream genes have been predicted to experience stronger evolutionary constraint than downstream genes because their control on flux through the pathway and because of the number of products that require their activity [69]. Indeed, previous studies of anthocyanin genes have found evidence for this positional effect, where upstream genes generally evolve more slowly due to differences in the selective constraints [50, 69, 70].

While our study found significant variation in rates of evolution across the genes, we did not find that most upstream genes experienced the highest constraint. *Chi* was the most rapidly evolving gene with the highest dN/dS ratio among the three (Fig. 3, Additional file 1: Figure S1) despite the fact that it occupies the most upstream position. Partitioned analyses of the three gene dataset showed that the dN/dS ratio for *Chi* is significantly higher than for *F3h*, and statistically indistinguishable from that for *Dfr* (Additional file 4: Table S3). This high rate of evolution for *Chi* may be related to several unique aspects of its function and evolutionary history. First, unlike the other two loci, *Chi* has at least two closely related duplicates [47, 48], which may allow for reduced constraint on the individual copies. Second, the reaction catalyzed by CHI, namely the cyclization of chalcone into naringenin, can occur spontaneously (although inefficiently) and thus is likely to occur in cells where CHS is active even if CHI is not present [71, 72]. These factors may help to explain why *Chi* evolves as quickly as the downstream genes like *Dfr*, a result also found in Rausher et al. [69]. Future studies examining the full pathway (Fig. 2) would clarify the extent to which the pathway position predicts rate variation and whether indeed *Chi* is a strong outlier among upstream genes.

## Conclusions

On a phylogenetic scale, trait losses have commonly been associated with molecular decay of the underlying pathway [27, 30, 73]. However, such decay is expected to be mitigated when the genes of the pathway are required for multiple functions [74]. In the case of floral anthocyanin pigmentation, our study shows that the core genes of the anthocyanin pigmentation pathway have been conserved through repeated losses of floral pigmentation, a result that likely reflects the importance of these genes for functions beyond flower color. The conservation of these genes preserves the potential for regain of flower color following loss as observed in *Iochroma* (Fig. 2) and other flowering plant clades [41].

Despite this overall structural conservation, we identify patterns consistent with relaxed selective constraint in two of the three pathway genes (*Chi* and *F3h*) following losses of floral pigmentation. Given that the upstream products of these two genes, flavones and flavonols, are often produced in flowers and leaves [38], the elevated dN/dS ratios we observe are not likely to indicate a decrease in functionality of the enzymes. Instead, this molecular evolutionary pattern may be a consequence of changes in the expression of these genes. Specifically, reduced gene expression is well known to result in higher rates of protein evolution due to relaxed selection for translational efficiency and robustness [75, 76]. Although it is not known whether losses of floral pigmentation in Iochrominae are associated with reduced expression of *Chi* and *F3h*, such downregulation has been documented in other taxa [77]. Ongoing studies in the Iochrominae system aimed at integrating analyses of molecular evolution, gene expression, and flavonoid production along the phylogeny will contribute to a better understanding of both the causes and consequences of phenotypic transitions.

## Methods

### Dataset construction

We sequenced *Chi-A*, *F3h,* and *Dfr* from a total of 22 Iochrominae species (shown in Fig. 1). These included all 7 species that lack floral anthocyanin pigmentation and 15 species that produce floral anthocyanins. The genes were amplified from floral bud cDNA prepared from greenhouse or field-collected material. Briefly, RNA was extracted using the Spectrum Total RNA extraction kit (Sigma-Aldrich, MO), and DNA removed using an on-column DNAse digestion (Qiagen, Netherlands). cDNA synthesis was carried out using 1ug of RNA and the SuperScriptII Reverse Transcription kit (Life Technologies, CA). *Chi, F3h,* and *Dfr* were amplified using primers designed from previous studies [59, 61]. Products were directly sequenced in both directions, assembled, and edited using Geneious 7.1.5. Voucher specimens and accession numbers for all sequences are provided in Additional file 2: Table S1. Sequences from six outgroup taxa were retrieved from Genbank or Solgenomics (solgenomics.net); these included five from across Solanaceae (*Nicotiana benthamiana*, *Solanum tuberosum*, *S. lycopersicum*, *Capsicum annuum* and *Petunia hybrida*) and one from the sister family Convolvulaceae (*Ipomoea purpurea*) (Fig. 1, Additional file 2: Table S1).

### Estimating rates of amino acid substitution

Codon-based maximum likelihood methods were implemented using the codeml program in PAML4.7a [78]. These methods estimate the strength and nature of selection acting on codon sites by calculating the ratio of non-synonymous to synonymous mutations (dN/dS or ω), where *ω* < 1 suggests purifying selection, *ω* = 1 indicates neutral evolution, and *ω* > 1 indicates positive selection. Variation in ω can be partitioned across genes, across sites within a gene, and across branches to test specific hypotheses. We first examined the hypothesis that flower color transitions (specifically losses of floral anthocyanins) have altered selective pressures on anthocyanin genes using branch models. These models allow for ω to differ between background (pigmented) and foreground (unpigmented) branches [79]. For significance testing, the two ratio branch models for each gene were compared to a single ratio model using likelihood ratio tests. We also considered branch models in which we divided the unpigmented lineages into two sets (e.g., separate ω values for unpigmented lineages in the ingroup versus the outgroup), and used the same likelihood ratio tests for model comparison. Observing that the ω values from the single ratio models varied across genes, we conducted an additional set of analyses, grouping the genes into all possible pairs to test differences in ω across the genes (Models “C” and “E”, [80]).

Within the same maximum likelihood framework, we used branch-site models to test for positive selection acting on the anthocyanin genes in unpigmented lineages. For these analyses, the null model assumes four site classes [81]. Class 0 includes sites that are conserved (*0 < ω* < 1) across all branches. Class 1 includes sites that evolve neutrally (*ω* = 1) across all branches. Class 2a contains sites that are conserved in the background, but become neutral in the foreground. Class 2b contains sites that evolve neutrally in the background, and are restricted to neutral in the foreground. The alternative model is similar, but allows for positive selection by permitting ω to vary freely in the foreground branches of classes 2a and 2b. These branch-site models also include a Bayes Empirical Bayes (BEB) procedure for the identification of sites under positive selection [52]. We considered sites to be positively selected if the posterior probability was greater than 0.95.

As the branch-site models are relatively parameter-rich, we also fit sites models for each gene, which are designed to detect positively selected sites across the entire tree. We conducted two sets of model comparisons for each gene, M1a versus M2a and M7 versus M8 [52, 82]. The M1a model has two site classes, one conserved (*0 <* ω < 1) and one neutral (*ω* = 1), while the M2a model has an extra category of positively selected sites (*ω* > 1). The M7 model describes variation in ω as a beta distribution between 0 and 1 with parameters estimated from the data. The distribution is discretized into 10 equally spaced categories [52]. The M8 model adds an extra site category where positive selection is permitted (*ω* ≥1).

### Analysis of physiochemical changes following losses of pigmentation

As a alternative approach to detecting changes in selection pressure associated with losses of pigmentation, we used the program TreeSAAP 3.2 [53] to examine the physiochemical effects of amino acid substitutions along unpigmented branches. TreeSAAP identifies positively selected sites by comparing the magnitude of the observed physiochemical changes (reconstructed along branches with PAML) to an expected distribution assuming random amino acid replacements [54]. The program examines 31 physiochemical properties, ranging from polarity to molecular weight, which are important for determining the structure and function of the protein. For each property, the effect of a substitution is assigned to a category from 1 to 8, with categories 6 through 8 being considered radical. Significance is determined by a goodness-of-fit test comparing the distribution of observed and expected effects [83]. For these analyses, we used a sliding window length of 20 sites and considered only sites with effects in the 6 to 8 magnitude range as radical. We examined only substitutions within in the portion of the protein known to contribute to its three-dimensional structure. To do this, Solanaceae sequences were aligned with known crystal structures for DFR [84] and CHI [72], viewed in the Swiss PDB viewer [85].

## Ethics

Not applicable.

## Consent to publish

Not applicable.

## Availability of data and materials

All sequence data has been submitted to Genbank via the following accession numbers: KT898394-KT898451. Sequence alignments have been placed on Dryad at doi:10.5061/dryad.49rs8.

## Additional files

Additional file 1: Figure S1. Gene trees for *Chi*, *F3h* and *Dfr*. (DOCX 88 kb) Additional file 2: Table S1. Source information and Genbank numbers for sequences. (DOCX 25 kb) Additional file 3: Table S2. Maximum likelihood tests of selection on *Chi*, *F3h*, and *Dfr*. (DOCX 26 kb) Additional file 4: Table S3. Comparisons of dN/dS across genes, on *Chi*, *F3h*, and *Dfr.* (DOCX 15 kb) Additional file 5: Table S4. Amino acid substitutions with significant physiochemical effects along unpigmented and pigmented branches. (DOCX 28 kb)

## Abbreviations

CHI: chalcone isomerase
DFR: dihydroflavonol reductase
F3H: flavanone-3-hydroxylase
