# Supplementary material for: Molecular evolution of anthocyanin pigmentation genes following losses of flower color
Source: BMC Evol Biol. 2016 May 10;16:98. doi: 10.1186/s12862-016-0675-3 (PMC4862180; doi:10.1186/s12862-016-0675-3)
Supplement: Additional file 1: Figure S1. — Gene trees for Chi, F3h and Dfr. (DOCX 88 kb) [file 12862_2016_675_MOESM1_ESM.docx]

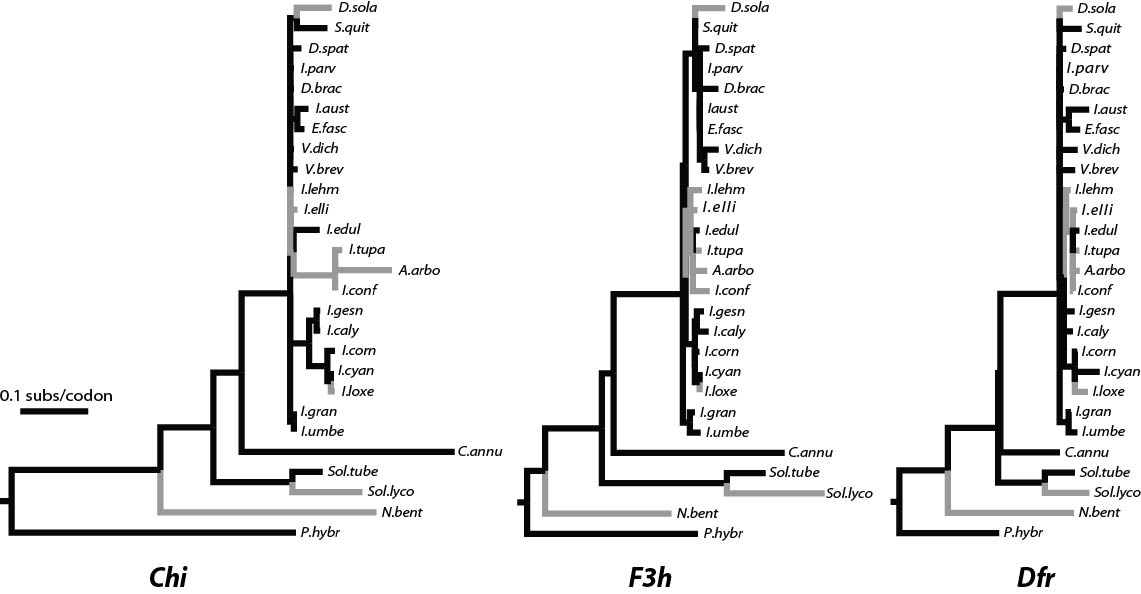
**Fig. S1.** Gene trees for *Chi*, *F3h* and *Dfr*. Branch lengths from PAML are in nucleotide substitutions per codon, estimated under the free ratio model. Taxon names are abbreviated to the first letter of the genus (except for Solanum abbreviated “Sol”) and the first four letters of the specific epithet. See Fig. 1 for full taxon names. Lineages with pigmentation are represented with black lines and those without with gray line.
