# Supplementary material for: Molecular evolution of anthocyanin pigmentation genes following losses of flower color
Source: BMC Evol Biol. 2016 May 10;16:98. doi: 10.1186/s12862-016-0675-3 (PMC4862180; doi:10.1186/s12862-016-0675-3)
Supplement: Additional file 2: Table S1. — Source information and Genbank numbers for sequences. (DOCX 25 kb) [file 12862_2016_675_MOESM2_ESM.docx]

TABLE S1. Source information and genbank numbers for sequences. For most of the species, the accessions are listed with reference to herbarium vouchers (collector, collection number, herbarium). The exceptions are the Solanaceae collection at Nijmegen (http://www.ru.nl/bgard/databases/solanaceae-database/), which are listed by accession numbers and seed purchased from Kartuz Greenhouses, Vista, CA. In cases where the voucher was representative of multiple individuals (e.g., greenhouse grown plants from seeds, or individuals in a population), the individual number (e.g., #1) is given. Genbank numbers are listed for the outgroup taxa except for *Nicotiana benthamiana*, in which case the scaffold numbers from the genome build are given (https://solgenomics.net).

| **SPECIES** | **VOUCHER**  **(Herbaria) Individual**  **GENBANK ACCESSION NO.** | | |
| --- | --- | --- | --- |
|  | *Dfr* | *F3h* | *Chi* |
| *Acnistus arborescens* | Bohs 2428 (UT) #4  KT898450 | Bohs 2428 (UT) #4  KT898406 | Bohs 2428 (UT) #4  KT898428 |
| *Dunalia brachyacantha* | Nee & Bohs 50811 (NY) #2  JN593319 | Nee & Bohs 50811 (NY) #2  KT898403 | Nee & Bohs 50811 (NY) #2  KT898430 |
| *Dunalia solanacea* | Smith 211 (MO) #A  JN593320 | Smith 211 (MO) #A  KT898396 | Smith 211 (MO) #A  KT898436 |
| *Dunalia spathulata* | Smith 455 (MO) #1  KT898440 | Smith 455 (MO) #1  KT898415 | Smith 455 (MO) #1  KT898429 |
| *Eriolarnyx fasciculata* | Smith 408 (F) #I  KT898447 | Smith 408 (F) #I  KT898395 | Smith 408 (F) #I  KT898426 |
| *Iochroma australe* | Nijmegen 904750145 #1  KT898438 | Nijmegen 904750145 #1  KT898397 | Nijmegen 904750145 #1  KT898427 |
| *Iochroma calycinum* | Smith 471 (F)  JN593314.1 | Smith 471 (F)  KT898394 | Smith 471 (F) #J  KT898417 |
| *Iochroma confertiflorum* | Smith 237 (MO)  KT898443 | Smith 334 (F) #A  KT898404 | Smith 334 (F) #A  KT898418 |
| *Iochroma cornifolium* | Smith 523 (MO)  KT898441 | Smith523 (MO)  KT898399 | Smith523 (MO)  KT898420 |
| *Iochroma cyaneum* | Smith 265 (WIS) #4  GU595064.1 | Smith 265 (WIS) #4  KT898413 | Smith 265 (WIS) #4  KT898421 |
| *Iochroma edule* | Smith 359 (MO) #2  KT898445 | Smith 359 (MO) #2  KT898401 | Smith 359 (MO) #2  KT898423 |
| *Iochroma ellipticum* | Jaeger 625 (CDS) #1  KT898446 | Jaeger 625 (CDS) #1  KT898414 | Jaeger 625 (CDS) #1  KT898425 |
| *Iochroma gesnerioides* | Nijmegen 934750129 #2  GU595063.1 | Nijmegen 934750129 #2  KT898408 | Nijmegen 934750129 #2  KT898437 |
| *Iochroma grandiflorum* | Kartuz 51862 #1  KT898442 | Kartuz 51862 #1  KT898411 | Kartuz 51862 #1  KT898432 |
| *Iochroma loxense* | Smith 220 (MO) #5  JN593313, JN593312 | Smith 235 (MO) #5  KT898409 | Smith 235 (MO) #5  KT898422 |
| *Iochroma parvifolium* | Smith 303 (MO)  JN593318.1 | Smith 304 (MO) #A  KT898405 | Smith 304 (MO) #A  KT898431 |
| *Iochroma squamosum* | Smith 330 (MO) #F  KT898448 | Smith 330 (MO) #F  KT898412 | Smith 330 (MO) #F  KT898434 |
| *Iochroma tupayachianum* | Smith 519 (MO) #C  KT898449 | Smith 519 (MO) #C  KT898398 | Smith 519 (MO) #C  KT898419 |
| *Iochroma umbellatum* | Smith 505 (MO)  JN593321, JN593322 | Smith 360 (MO) #u5  KT898402 | Smith 360 (MO) #u5  KT898433 |
| *Saracha quitensis* | Smith 257 (MO)  KT898444 | Smith 257 (MO)  KT898410 | Smith 257 (MO)  KT898416 |
| *Vassobia breviflora* | Nijmegen 904750332 #2  KT898451 | Nijmegen 904750332 #2  KT898407 | Nijmegen 904750332 #2  KT898424 |
| *Vassobia dichotoma* | Smith 440 (MO) #C  KT898439 | Smith 440 (MO) #C  KT898400 | Smith 440 (MO) #C  KT898435 |
| *Ipomoea purpurea* | AF028601 | U74081 | AF028238 |
| *Petunia hybrida* | KC140107 | AF022142 | X14589 |
| *Nicotiana benthamiana* | NibenScf51239 | Niben044Scf00002714Ctg0 | Niben.V0.4.2.Scf28382_14.0 |
| *Solanum lycopersicum* | NM001247479 | XM004232440 | NM_001320711 |
| *Solanum tuberosum* | AF449422 | AY102035. | XM006348549 |
| *Capsicum annuum* | JN885196 | JN808445 | FJ705843 |
