# Supplementary material for: Molecular evolution of anthocyanin pigmentation genes following losses of flower color
Source: BMC Evol Biol. 2016 May 10;16:98. doi: 10.1186/s12862-016-0675-3 (PMC4862180; doi:10.1186/s12862-016-0675-3)
Supplement: Additional file 3: Table S2. — Maximum likelihood tests of selection on Chi, F3h, and Dfr. (DOCX 26 kb) [file 12862_2016_675_MOESM3_ESM.docx]

**TABLE S2.** Maximum likelihood tests of selection on Chi, F3h, and Dfr, implemented in PAML 4.7a. *(*a) Likelihoods of the free ratio and the 2-ꙍ models were compared using degrees of freedom equal to the difference in the number of parameters between the models. Likelihoods of the 3-ꙍ models were compared with 1-ꙍ (and 2-ꙍ, results not shown). Significant improvements in likelihood are indicated with asterisks: **p<0.001, *p<0.05. For 2-ꙍ models, the dN/dS ratio (ꙍ) is given with a subscript ‘p’ for lineages with floral anthocyanin pigments and ‘n’ for lineages without. The 54 ratios (for the 54 branches) of the free model are not shown. For 3-ꙍ models, ꙍ is given with a subscript ‘p’ for lineages with floral anthocyanin pigments, ‘in’ for Iochrominae lineages without, ‘out’ for outgroup lineages without, ‘tac’ for the subclade *I. tupayachianum, A. arborescens, I. confertiflorum* without, and ‘w’ for the remaining lineages without floral anthocyanin pigements excluding ‘tac’. b) Comparisons of two branch sites models (Yang et al. 2005). The alternate model places sites into one of four categories: ꙍ less than 1 in both background (pigmented) and foreground (unpigmented) lineages (‘pur./pur.’), ꙍ equal to 1 in both (‘neu./neu.’), ꙍ less than 1 in the background but neutral or positively selected in the foreground (‘pur./neu.’), and ꙍ equal to 1 in background but positively selected in the foreground (‘neu./pos.’). This model is compared against the null model where the fourth category of sites is fixed at ꙍ equal to 1 in both (‘neu./neu.’). (c) Comparisons of sites models M1a vs. M2a (allows an additional site class for positive selection), and M7 vs. M8 (allows an additional site class for positive selection) (Wong et al. 2004, Yang et al. 2005).

1. Branch models

|  | Model | No. parameters | ln *L* | ꙍ |
| --- | --- | --- | --- | --- |
| *Chi* | 1-ꙍ | 1 | -3126.75 | ꙍ=0.243 |
|  | Free | 54 | -3090.90* |  |
|  | 2-ꙍ | 2 | -3121.13**** | ꙍ_p_=0.185, ꙍ_n_=0.377 |
|  | 3-ꙍ | 3 | -3120.36 * | ꙍ_p_=0.185, ꙍ_in_=0.429, ꙍ_out_=0.271 |
|  | 3-ꙍ | 3 | -3120.98 * | ꙍ_p_= 0.185, ꙍ_w_=0.394, ꙍ_tac_=0.312 |
| *F3h* | 1-ꙍ | 1 | -4147.97 | ꙍ= 0.0932 |
|  | Free | 54 | -4114.83 |  |
|  | 2-ꙍ | 2 | -4145.35*** | ꙍ_p_ = 0.0792, ꙍ_n_ = 0.128 |
|  | 3-ꙍ | 3 | -4144.79 * | ꙍ_p_=0.0794, ꙍ_in_=0.116, ꙍ_out_=0.174 |
|  | 3-ꙍ | 3 | -4145.15 | ꙍ_p_=0.0792, ꙍ_w_=0.132, ꙍ_tac_=0.0913 |
| *Dfr* | 1-ꙍ | 1 | -4192.80 | ꙍ=0.214 |
|  | Free | 54 | -4161.67 |  |
|  | 2-ꙍ | 2 | -4192.77 | ꙍ_p_=0.211, ꙍ_n_=0.222 |
|  | 3-ꙍ | 3 | -4190.023968 | ꙍ_p_=0.211, ꙍ_in_=0.173, ꙍ_out=_0.505 |
|  | 3-ꙍ | 3 | -4192.684169 | ꙍ_p_=0.211, ꙍ_w=_0.218, ꙍ_tac_=0.319 |

1. Branch-Sites models

|  | Model | ln *L* | Proportion of Sites | | | |
| --- | --- | --- | --- | --- | --- | --- |
|  |  |  | **pur./pur.** | **neu./neu.** | **pur./neu.** | **neu./neu. or pos.** |
| *Chi* | null | -3080.41 | 0.661 | 0.181 | 0.124 | 0.0340 |
|  | alt | -3080.24 | 0.683 | 0.184 | 0.105 | 0.0282 |
| *F3h* | null | -4109.67 | 0.87927 | 0.0679 | 0.0490 | 0.00379 |
|  | alt | -4109.67 | 0.87927 | 0.0679 | 0.0490 | 0.00379 |
| *Dfr* | null | -4133.87 | 0.738 | 0.195 | 0.0523 | 0.0138 |
|  | alt | -4133.87 | 0.676 | 0.195 | 0.0523 | 0.0138 |

1. Sites models

|  | Model | ln *L* | Proportion of Sites | | |
| --- | --- | --- | --- | --- | --- |
|  |  |  | **ꙍ<1** | **ꙍ=1** | **ꙍ>1** |
| *Chi* | M1a | -3086.16 | 0.746 | 0.254 | - |
|  | M2a | -3086.16 | 0.746 | 0.194 | 0.0602 |
|  | M7 | -3083.17 | - | - | - |
|  | M8 | -3082.69 | - | - | 0.00465 |
| *F3h* | M1a | -4112.39 | 0.925 | 0.0750 | - |
|  | M2a | -4112.39 | 0.925 | 0.0750 | 0 |
|  | M7 | -4098.99 | - | - | - |
|  | M8 | -4098.80 | - | - | 0.00915 |
| *Dfr* | M1a | -4134.63 | 0.785 | 0.215 | - |
|  | M2a | -4134.63 | 0.785 | 0.184 | 0.0309 |
|  | M7 | -4133.41 | - | - | - |
|  | M8 | -4130.02* | - | - | 0.0225 |
