# Supplementary material for: Molecular evolution of anthocyanin pigmentation genes following losses of flower color
Source: BMC Evol Biol. 2016 May 10;16:98. doi: 10.1186/s12862-016-0675-3 (PMC4862180; doi:10.1186/s12862-016-0675-3)
Supplement: Additional file 4: Table S3. — Comparisons of dN/dS across genes, on Chi, F3h, and Dfr. (DOCX 15 kb) [file 12862_2016_675_MOESM4_ESM.docx]

**TABLE S3.** dN/dS comparisons across genes. Model C allows for different codon frequencies and substitution rates for each genes, but fits a single dN/dS ratio (ꙍ). Model E estimates a separate ꙍ for each partition (listed in the Table in the order of the genes listed). **Significant increase in likelihood (p<0.001).

| Partition | Model | ln *L* | ꙍ |
| --- | --- | --- | --- |
| All genes (*Chi*, *F3h*, *Dfr*) | C | -11937.09 | 0.171 |
|  | E | -11902.17** | 0.242, 0.092, 0.224 |
| *Chi*, *F3h* | C | -7356.19 | 0.147 |
|  | E | -7330.80** | 0.244, 0.093 |
| *Chi*, *Dfr* | C | -7688.92 | 0.231 |
|  | E | -7687.12 | 0.240, 0.223 |
| *F3h*, *Dfr* | C | -7685.62 | 0.120 |
|  | E | -7677.26** | 0.093, 0.163 |
