# Supplementary material for: Molecular evolution of anthocyanin pigmentation genes following losses of flower color
Source: BMC Evol Biol. 2016 May 10;16:98. doi: 10.1186/s12862-016-0675-3 (PMC4862180; doi:10.1186/s12862-016-0675-3)
Supplement: Additional file 5: Table S4. — Amino acid substitutions with significant physiochemical effects along unpigmented and pigmented branches. (DOCX 28 kb) [file 12862_2016_675_MOESM5_ESM.docx]

TABLE S3. Amino acid substitutions with significant physiochemical effects along (A) unpigmented and (B) pigmented branches. Tests implemented in TreeSAAP 3.2.

| (A) |  |  |  |  |
| --- | --- | --- | --- | --- |
| Gene | Location | Site (Substitution) | Property affected |  |
| *Chi* | *N.bent* terminal branch | 96 (Thr🡪Ile) | Equilibrium constant^1^ |  |
|  |  | 168 (Ser🡪Ile) | Chromatographic index^2^, Equilibrium constant |  |
|  |  | 214 (Glu🡪Ser) | Alpha helical tendencies^3^ |  |
|  | *Sol.lyco* terminal branch | 30 (Thr🡪Met) | Alpha helical tendencies |  |
|  |  | 132 (Arg🡪Met) | Chromatographic index |  |
|  | *I.squa* terminal branch | 38 (Thr🡪Met) | Alpha helical tendencies |  |
| *Dfr* | *Sol.lyco* terminal branch | 21 (Cys🡪Trp) | Chromatographic index |  |
| \| (B) \|  \| \|  \| \|  \| \| --- \| --- \| --- \| --- \| --- \| --- \| \| Gene \| Location \| Site (Substitution) \| \| Property affected \| \| \| | | | | |

| *Chi* | *Ipo.purp* terminal branch | 5 (Ala🡪Pro) | Alpha helical tendencies |
| --- | --- | --- | --- |
|  |  | 11 (Gln🡪Lys) | Isoelectric point^4^ |
|  |  | 29 (Asn🡪Lys) | Isoelectric point |
|  |  | 38 (Asn🡪Ala) | Alpha helical tendencies |
|  |  | 126 (Thr🡪Met) | Alpha helical tendencies |
|  |  | 133 (Gln🡪Lys) | Isoelectric point |
|  |  | 146 (Thr🡪Met) | Alpha helical tendencies |
|  |  | 176 (Thr🡪Ile) | Equilibrium constant |
|  |  | 210 (Glu🡪Val) | Chromatographic index |
|  |  | 217 (Lys🡪Ile) | Chromatographic index; Equilibrium constant |
|  | node#29 🡪 node#30 | 143 (Arg🡪Gln) | Isoelectric point |
|  | *P.hybr* terminal branch | 133 (Gln🡪Arg) | Isoelectric point |
|  |  | 162 (Ser🡪Leu) | Chromatographic index |
|  | *N.bent* terminal branch | 96 (Thr🡪Ile) | Equilibrium constant |
|  | node#30 🡪 node#31 | 79 (Asp🡪His) | Isoelectric point |
|  |  | 210 (Glu🡪Lys) | Isoelectric point |
|  | node#31 🡪 node#32 | 6 (Ser🡪Leu) | Chromatographic index |
|  | *Sol.lyco* terminal branch | 132 (Arg🡪Met) | Chromatographic index; Isoelectric point |
|  | *C.annu* terminal branch | 75 (Glu🡪Lys) | Isoelectric point |
|  | node#33 🡪 node#34 | 30 (Thr🡪Ile) | Equilibrium constant |
|  |  | 206 (Cys🡪Phe) | Chromatographic index |
|  | *V.dich* terminal branch | 164 (Thr🡪Met) | Alpha helical tendencies |
| *F3h* | *Ipo.purp* terminal branch | 4 (Pro🡪Val) | Solvent accessible reduction ratio^5^ |
|  |  | 50 (Glu🡪Val) | Solvent accessible reduction ratio |
|  |  | 59 (Gly🡪Val) | Solvent accessible reduction ratio |
|  |  | 351 (Gln🡪Ile) | Solvent accessible reduction ratio |
|  | *P.hybr* terminal branch | 354 (Asp🡪Val) | Solvent accessible reduction ratio |
|  | node#31 🡪 node#32 | 50 (Asp🡪Val) | Solvent accessible reduction ratio |
| *Dfr* | *Ipo.purp* terminal branch | 35 (Met🡪Lys) | Solvent accessible reduction ratio |
|  |  | 76 (Asp🡪Val) | Solvent accessible reduction ratio |
|  |  | 79 (Val🡪Glu) | Solvent accessible reduction ratio |
|  |  | 148 (Glu🡪Pro) | Solvent accessible reduction ratio |
|  | node#33 🡪 node#34 | 18 (Pro🡪Arg) | Solvent accessible reduction ratio |
|  | *I.cyan* terminal branch | 18 (Arg🡪Trp) | Solvent accessible reduction ratio |
|  | *I.aust* terminal branch | 79 (Val🡪Glu) | Solvent accessible reduction ratio |

Woolley et al. 2003, and references therein. ^1^“Equilibrium constant with reference to the ionization property of the -COOH group” of a residue ^2^“…specifies characteristic migration rate in a solvent-absorbant system” ^3^helix propensity ^4^pH at neutrality ^5^ratio of solvent accessible surface area
